# Laparoscopic surgery contributes more to nutritional and immunologic recovery than fast-track care in colorectal cancer

**DOI:** 10.1186/s12957-015-0445-5

**Published:** 2015-02-04

**Authors:** Dong Xu, Jun Li, Yongmao Song, Jiaojiao Zhou, Fangfang Sun, Jianwei Wang, Yin Duan, Yeting Hu, Yue Liu, Xiaochen Wang, Lifeng Sun, Linshan Wu, Kefeng Ding

**Affiliations:** Department of Surgical Oncology, The Second Affiliated Hospital, Zhejiang University, School of Medicine, Zhejiang, China; Cancer Institute, Key Laboratory of Cancer Prevention and Intervention, China National Ministry of Education, Key Laboratory of Molecular Biology in Medical Sciences, The Second Affiliated Hospital, Zhejiang University, School of Medicine, 88 Jiefang Road, Hangzhou, 310009 Zhejiang Province China

**Keywords:** Colorectal surgery, Rehabilitation, Colorectal neoplasm, Fast-track care, Laparoscopy

## Abstract

**Background:**

Many clinical trials had repeatedly shown that fast-track perioperative care and laparoscopic surgery are both preferred in the treatment of colorectal cancer. But few studies were designed to explore the diverse biochemical impacts of the two counterparts on human immunologic and nutritional status.

**Methods:**

Ninety-two cases of colorectal cancer patients meeting the inclusion criteria were randomized to four groups: laparoscopy with fast-track treatment (LAFT); open surgery with fast-track treatment (OSFT); laparoscopy with conventional treatment (LAC); open surgery with conventional treatment (OSC). Peripheral blood tests including nutritional factors (albumin, prealbumin, and transferrin), humoral immunologic factors (IgG, IgM, and IgA), and cellular immunologic factors (T and NK cells) were evaluated. Blood samples were collected preoperatively (baseline) and 12 and 96 h after surgery (indicated as POH12 and POH96, respectively).

**Results:**

Albumin, transferrin, prealbumin, and IgG levels were the highest in the LAFT group for both POH12 and POH96 time intervals. Repeated measures (two-way ANOVA) indicated that the difference of albumin, transferrin, and IgG level were attributed to surgery type (*P* < 0.05) and not perioperative treatment (*P* > 0.05). Only in the laparoscopy-included groups, the relative albumin and IgG levels of POH96 were obviously higher than that of POH12.

**Conclusion:**

Laparoscopic surgery accelerated postoperative nutrition and immune levels rising again while fast-track treatment retarded the drop of postoperative nutrition and immune levels. Laparoscopic surgery might play a more important role than fast-track treatment in the earlier postoperative recovery of nutritional and immunologic status. Combined laparoscopic surgery with fast-track treatment provided best postoperative recovery of nutrition and immune status. These results should be further compared with the clinical outcomes of our FTMDT trial (clinicaltrials.gov: NCT01080547).

## Background

In the past few years, the introduction of two feasible treatments, the fast-track recovery program after surgery [1] and laparoscopic surgery, has been proved to significantly benefit patients. Many clinical trials have indicated improved recovery, shortening of hospital stay, decreased duration of paralytic ileus, and reduction in deterioration of vital organs when compared with patients undergoing conventional perioperative care and open surgery [2-4]. But many of the above trials only concerned the clinical impact of the fast-track care and laparoscopy, respectively; extremely few studies were designed to explore the biochemical impacts on human immunity and nutrition when combined fast-track care with laparoscopic techniques. And it still remained unclear that how much of the clinical benefit was due to laparoscopy and how much was an effect of different perioperative care [5].

LAFA group were concerned for a long time about the combined treatment of laparoscopic colostomy and fast-track care. According to the results from LAFA trial, the optimal combination of treatment for colon cancer patients is a laparoscopic surgery combined with a fast-track perioperative care [6,7]. And among colorectal patients, the EnROL trial also indicated laparoscopic surgery confers a significant clinical benefit versus open surgery embedded in an enhanced recovery program [8]. According to our previous study, we have registered and reported a 2 × 2 randomized controlled trial focusing on multi-discipline approach to implementation of a fast-track program (The Fast Track Multi-Discipline Treatment—FTMDT trial, clinicaltrials.gov: NCT01080547). The operable protocol of the FTMDT trial had been published [9], and the further research program concerned two directions were carried out. Direction 1 concerned more about the clinical parameters of patients such as hospitalization stay, return of bowel movement, etc. while direction 2 followed closely the detailed and biochemical impacts of the combined treatment of laparoscopic colostomy and fast-track perioperative treatments.

Albumin, prealbumin (PAB), and transferrin (TRF) are commonly called as “hepatic proteins” that are synthesized in the liver. By evaluating the levels of serum albumin, PAB, and TRF of patients undergoing colorectal surgery, we were able to access the status of postoperative nutrition and nutrition recovery [10-13]. Moreover, by evaluating the serum IgG, IgA, and IgM levels (representing humoral immunity) [14] and circulating T and NK cells (representing cellular immunity) [15] in different time intervals, we can also assess the levels of postoperative immune recovery. So in the present study, we collected and analyzed the nutritional and immunologic data in our FTMDT trial and aimed to investigate the exact biochemical roles of the two counterparts (laparoscopy and fast-track care) in the clinical context.

In this study, we aimed to evaluate prospectively the biochemical impacts of laparoscopic surgery and fast-tack care on postoperative nutrition and immune recovery among the colorectal patients. We are also earn to distinguish the exact role and weight of the two counterparts (laparoscopy and fast-track care) if beneficial data are obtained.

## Methods

### Study design

**Figure 1:**
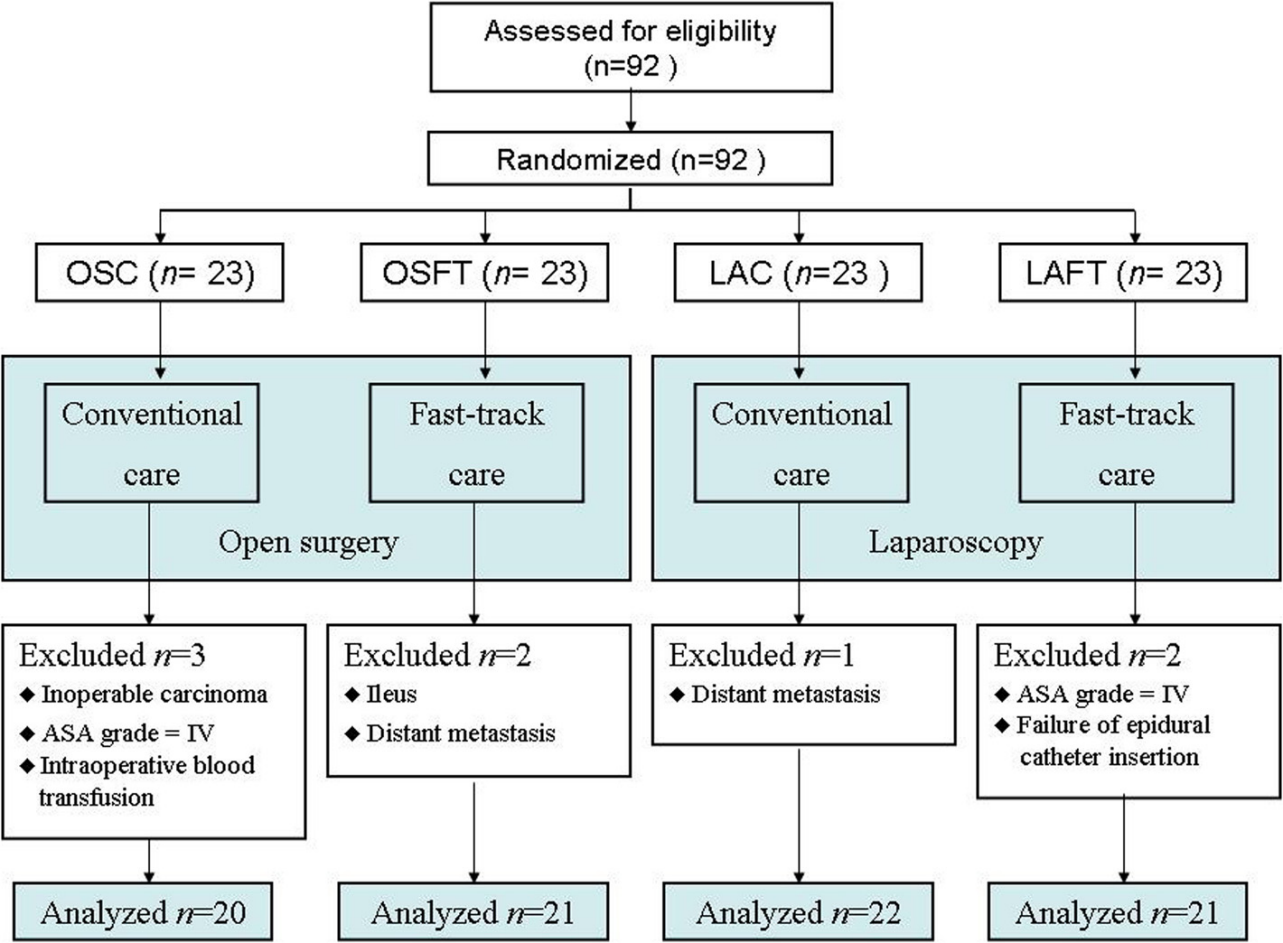
**Study flow.**

Our FTMDT trial is a randomized prospective and controlled study with 2 × 2 balanced factorial design. Patients were enrolled from December 2010 to December 2012. Patients were randomized into four treatment groups (1:1:1:1) by means of the randomized numbers generated by the SPSS 16.0. After written informed consent had been obtained, patients were randomized to laparoscopy or open surgery and to fast-track or conventional treatment (Figure 1). It generated four groups: (1) laparoscopy with fast-track treatment (LAFT); (2) open surgery with fast track treatment (OSFT); (3) laparoscopy with conventional treatment (LAC); (4) open surgery with conventional treatment (OSC).

### Eligibility

*Inclusion criteria*:

1. American Society of Anesthesiologists (ASA) grades I–III (no life-threatening systemic diseases)
2. Age ≥18 years old
3. With pathologically confirmed colon and upper rectal cancer.

*Exclusion criteria*:

1. Patients are younger than 18 years
2. ASA grade ≥ IV
3. Preoperative evidence of distant metastases
4. History of malignant disease
5. Tumors can be resected by endoscopic mucosal resection (EMR) or endoscopic submucosal dissection (ESD), bowel obstruction or perforation, and patients undergoing total colectomy, mid-low rectal cancer, and pregnancy.

### Ethics

This study was carried out according to the rules of the *Declaration of Helsinki* and the *CONSORT statement*. The independent medical ethics committee of the participating hospital approved the study protocol, with the approval number: 2010LSY No. 6. The study was registered under ClinicalTrials.gov: NCT01080547.

### Fast-track protocol

The fast-track treatment protocol for colorectal surgery was well established and reported previously [9]. The protocol contained oral carbohydrates before surgery, fluid restriction, body warming, early oral nutrition and early ambulation, and early removal of nasogastric tube. Patients were informed about the type of perioperative treatment but were blinded to the type of surgery. During the course of FTMDT trial, mechanical bowel preparation was routinely included in the perioperative treatments for all colorectal patients. We have described in detail the protocol of fast-track treatment previously.

### Measurements

Surgical information was carefully recorded in detail, including surgery types, operation time, and blood loss, et al. The surgery-associated complications were recorded postoperatively.

Peripheral blood tests include nutritional factors (albumin, prealbumin, and transferrin levels), humoral immunologic factors (circulating IgG, IgM, and IgA levels), and cellular immunologic factors (circulating T cells and NK cells). Peripheral blood samples were collected preoperatively (baseline) and 12 and 96 h after surgery (indicated as POH12 and POH96, respectively). All blood samples were taken from peripheral veins and were transported to the laboratory immediately.

### Immune status

#### Assessment of IgG, IgA, and IgM levels

Quantifications of IgG, IgA, and IgM levels were determined by Immunological Turbidity Kits for human IgG/IgA/IgM protein manufactured by Beijing Condor-Teco Medical Technology Co. Ltd. (Beijing, China).

#### Assessment of T and NK cell counts

The monoclonal antibodies (CD3-FITC/CD16 + CD56-PE and CD4-FITC/CD8-PE/CD3-PC5) used for flow cytometric quantification were purchased from Beckman Coulter, Inc. (Marseille, France). Fluorescent-activated cell sorting analysis was carried out on the BD FACScan flow cytometer (Becton, Dickinson and Company, San Jose, CA, USA) and a minimum of 10,000 cells were assessed for each detection. After flow cytometric sorting analysis, CD3+ populations indicate T lymphocytes, and CD3-/CD16+/CD56+ populations indicate NK lymphocytes.

### Nutrition status

#### Assessment of albumin (ALB), PAB, and TRF levels

Serum albumin, PAB, and TRF levels were detected using N Antiserum to Human Albumin/PAB/TRF Kits manufactured by Siemens Healthcare Diagnostics Products (Marburg, Germany).

### Statistical analysis

Data were tabulated on the Excel sheet (Excel 2007 for Windows; Microsoft Corporation, Redmond, WA, USA) and then were analyzed using SPSS 16.0 for Windows (SPSS, Chicago, IL, USA). Numerical variables were presented as the mean ± SD or percentage of the baseline level unless otherwise stated. ANOVA, chi-square, and Kruskal-Wallis tests were applied for data comparison when appropriate. The repeated measures (two-way ANOVA) were conducted for analysis of the 2 × 2 factorial design on initial data. *P* < 0.05 was considered statistically significant.

## Results

A total of 92 patients were randomized, and 84 patients finally finished the study (Figure 1). Twenty-one patients were randomized for LAFT group, 21 for OSFT, 22 for LAC, and 20 for OSC. The four groups were balanced with respect to patients’ characteristics, including sex, age, BMI, ASA grade, tumor staging, or type of surgery (Table 1).

**Table 1:** **Patients’ baseline characteristics**

**Characteristics**	**OSC group (** ***n*** **= 20)**	**OSFT group (** ***n*** **= 21)**	**LAC group (** ***n*** **= 22)**	**LAFT group (** ***n*** **= 21)**	***P***
Male sex (%)^a^	65.00	61.90	68.18	57.14	0.895
Age (years)^b^	58.0 ± 13.2	59.3 ± 12.5	60.8 ± 7.6	59.1 ± 9.8	0.871
Body mass index, (kg/m^2^)^b^	22.7 ± 3.0	22.7 ± 2.0	23.8 ± 3.2	23.2 ± 2.6	0.504
Type of surgery, *n* (%)^c^					0.946
Right hemicolectomy	6 (30.0%)	6 (28.6%)	6 (27.3%)	5 (23.8%)	
Left hemicolectomy	3 (15.0%)	4 (19.0%)	3 (13.6%)	3 (14.3%)	
Sigmoidectomy	4 (20.0%)	3 (14.3%)	4 (18.2%)	4 (19.0%)	
Dixon operation	7 (35.0%)	8 (38.1%)	9 (40.9%)	9 (42.9%)	
TNM staging, *n* (%)^c^					0.995
I	2 (10%)	3 (14.3%)	2 (9.1%)	4 (19.0%)	
II	10 (50%)	8 (38.1%)	11 (50%)	7 (33.4%)	
III	8 (40%)	10 (47.6%)	9 (40.9%)	10 (47.6%)	
ASA (%)^a^					
Grade I or II	85.0	81.0	86.4	85.7	0.962
Operating time (minutes)^b^	137.7 ± 21.8	134.0 ± 23.8	193.1 ± 31.5	184.8 ± 43.4	0.000
Blood loss (ml)^b^	172.2 ± 77.1	177.5 ± 92.4	97.6 ± 53.0	104.7 ± 60.0	0.000
Incision length (cm)^b^	20.6 ± 2.0	20.9 ± 2.9	4.5 ± 0.8	4.3 ± 0.7	0.000

*ASA* indicates American Society of Anaesthesiologists.

Variables were expressed as the mean ± SD.

^a^Chi-square test.

^b^ANOVA test.

^c^Kruskal-Wallis test.

### Nutritional status

All postoperative values (presented in percentage from baseline) for nutritional status, as well as the mean values for POH12 and POH96 time intervals, are shown in Table 2.

**Table 2:** **Postoperative nutritional and immunologic status**

**Marker**	**Time**	**OSC**	**OSFT**	**LAC**	**LAFT**
*Albumin*	POH12	77.3 (62.3–95.9)	81.3 (57.7–107.5)	86.5 (64.9–100.3)	89.0 (66.0–133.8)
	POH96	75.9 (56.2–104.8)	80.4 (60.2–109.8)	89.5 (67.0–106.3)	97.3 (76.0–176.9)
	Mean	76.6 (11.2)	80.9 (12.9)	88.0 (10.4)	93.1 (20.6)
*Prealbumin*	POH12	76.0 (53.2–101.6)	75.3 (47.8–130.6)	78.8 (54.0–110.6)	80.4 (61.5–100.5)
	POH96	68.2 (38.8–124.1)	72.9 (32.6–110.2)	72.9 (33.4–146.6)	75.7 (47.2–110.1)
	Mean	72.1 (19.8)	74.1 (18.8)	75.9 (21.0)	78.1 (14.5)
*Transferrin*	POH12	79.5 (65.8–100.0)	81.8 (57.9–100.4)	87.4 (68.9–113.3)	89.3 (50.4–121.4)
	POH96	75.6 (53.1–92.8)	82.6 (71.7–113.6)	88.4 (50.5–123.8)	92.0 (62.3–135.2)
	Mean	77.6 (11.1)	82.2 (11.1)	87.9 (14.0)	90.7 (19.3)
*IgG*	POH12	82.3 (67.2–100.0)	84.1 (65.6–104.7)	87.6 (72.0–101.3)	88.2 (72.6–101.2)
	POH96	82.8 (67.7–114.4)	85.8 (61.8–123.1)	92.7 (70.8–109.0)	94.9 (80.1–121.7)
	Mean	82.6 (9.7)	85.0 (12.2)	90.2 (10.1)	91.6 (10.5)
*IgA*	POH12	84.7 (66.5–107.5)	85.8 (63.6–105.9)	88.6 (74.3–106.3)	88.7 (70.3–120.8)
	POH96	97.3 (82.7–169.0)	98.1 (66.9–124.6)	95.5 (73.3–111.6)	98.2 (80.1–115.7)
	Mean	91.0 (16.2)	92.0 (13.0)	92.1 (11.3)	93.4 (11.6)
*IgM*	POH12	76.9 (52.3–100.0)	79.0 (56.1–122.2)	85.4 (36.9–113.7)	87.6 (49.0–116.0)
	POH96	91.6 (86.5–101.2)	90.6 (61.3–179.2)	95.1 (34.5–271.2)	93.1 (64.6–145.9)
	Mean	84.3 (20.1)	84.8 (24.3)	90.2 (34.2)	90.3 (17.6)
*T cells*	POH12	88.5 (53.9–171.8)	87.9 (58.8–121.1)	84.6 (53.8–103.4)	86.8 (57.6–128.3)
	POH96	91.7 (70.2–128.5)	95.2 (60.8–130.6)	106.2 (57.5–152.9)	102.6 (79.5–126.0)
	Mean	90.1 (17.9)	91.6 (19.3)	95.4 (20.9)	94.7 (16.4)
*NK cells*	POH12	113.9 (43.5–314.3)	144.6 (56.2–401.9)	137.0 (90.2–246.0)	147.7 (39.8–324.3)
	POH96	88.9 (42.3–338.1)	83.8 (25.2–166.4)	102.1 (43.4–188.9)	97.2 (50.9–158.5)
	Mean	101.4 (68.3)	114.2 (74.4)	119.5 (44.4)	122.4 (57.2)

*POH12* indicates post-operation 12 h, *POH96* indicates post-operation 96 h, and *POH12* and *POH96* values are presented in percentage from baseline (preoperative value) for better comparison and range in parentheses.

*Mean* indicates mean values for POH12 and POH96 time intervals. Mean values are also presented in percentage from baseline and SD in parentheses.

**Figure 2:**
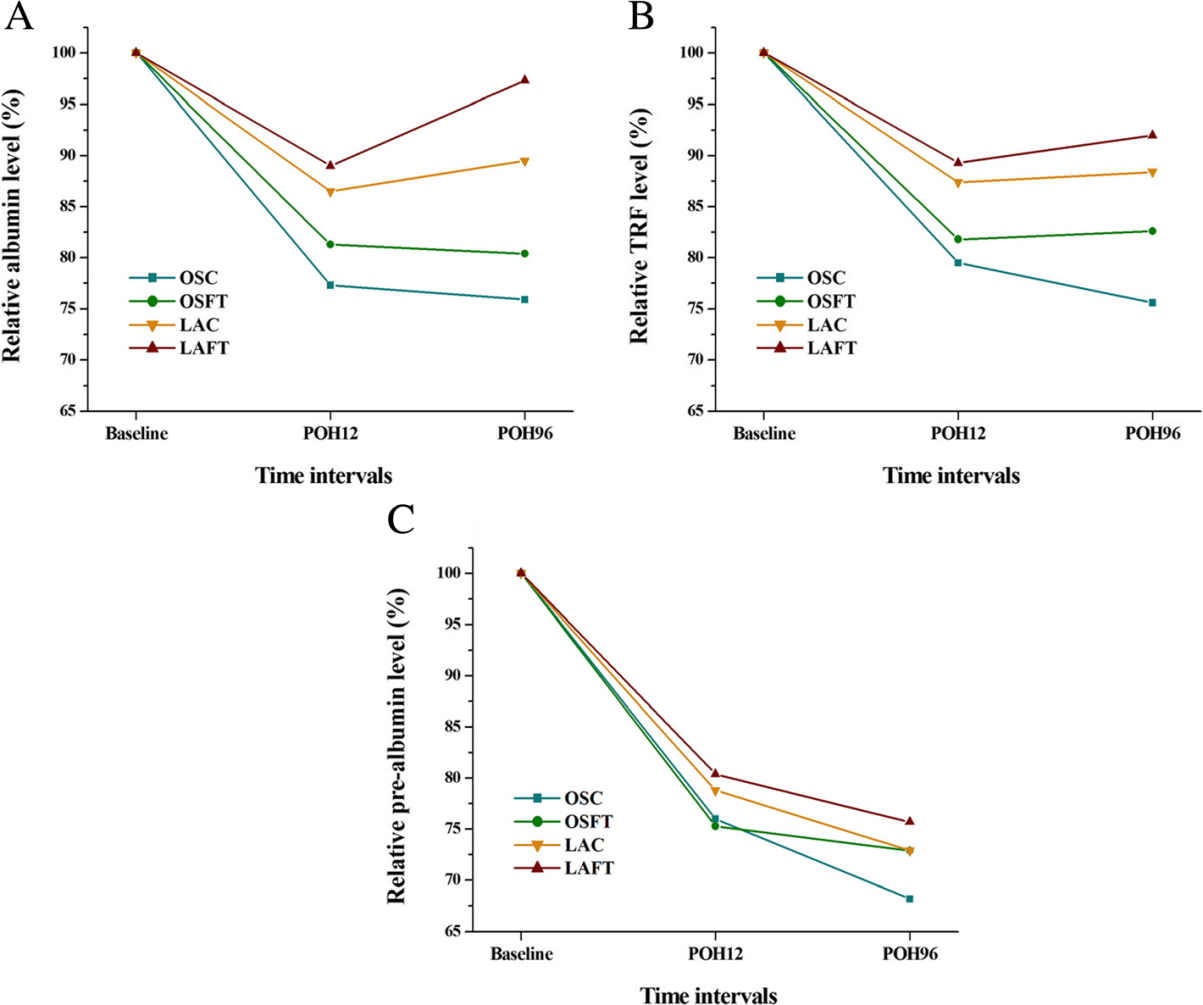
**Nutritional status of different time intervals in the four groups: albumin (A), TRF (B), prealbumin (C).**

As a result, Figure 2 shows three important points: 1) What is most interesting is that, only in the laparoscopy-included groups (LAFT and LAC groups), the albumin level of 96 h (POH96) was higher than that of 12 h (POH12), indicating the better potency of postoperative recovery of nutritional status; 2) In the fast-track care-included groups (OSFT and LAFT groups), the serum albumin level of POH12 and POH96 were both higher than that in only conventional care-included and same surgery-type groups (OSC and LAC groups), indicating that fast-track treatment retards the decrease of postoperative nutritional levels; 3) Albumin levels were highest in the LAFT group for both POH12 and POH96 time intervals.

Repeated measures (two-way ANOVA) indicated that the difference of albumin level can be attributed to surgery type and not perioperative treatment. No cross interaction was found between surgery type and perioperative treatment (Table 3).

**Table 3:** ***P*** **values of repeated-measures (two-way ANOVA) analysis**

**Marker**	**Variable**	***P***
*Albumin*	Surgery types	*0.000*
	Perioperative treatment types	*0.065*
	Cross interaction^a^	*0.696*
*Prealbumin*	Surgery types	*0.784*
	Perioperative treatment types	*0.671*
	Cross interaction	*0.768*
*Transferrin*	Surgery types	*0.002*
	Perioperative treatment types	*0.109*
	Cross interaction	*0.837*
*IgG*	Surgery types	*0.001*
	Perioperative treatment types	*0.419*
	Cross interaction	*0.603*
*IgA*	Surgery types	*0.503*
	Perioperative treatment types	*0.568*
	Cross interaction	*0.742*
*IgM*	Surgery types	*0.166*
	Perioperative treatment types	*0.902*
	Cross interaction	*0.601*
*T cells*	Surgery types	*0.306*
	Perioperative treatment types	*0.999*
	Cross interaction	*0.800*
*NK cells*	Surgery types	*0.542*
	Perioperative treatment types	*0.647*
	Cross interaction	*0.640*

^a^Cross interaction between surgery types (laparoscopy or open surgery) or perioperative treatment types (fast-track or conventional treatment).

TRF levels for both POH12 and POH96 time intervals were highest in the LAFT group and higher in the LAC group than the other two open surgery groups (Table 2; Figure 2B). Repeated measures (two-way ANOVA) indicated that the difference of TRF level can be attributed to surgery type and not perioperative treatment. No interaction was found between surgery type and perioperative treatment (Table 3).

Prealbumin levels were also the highest in the LAFT group for both POH12 and POH96 time intervals (Table 2; Figure 2C). But the following two-way ANOVA analysis that revealed no difference can be found between surgery type as well as perioperative treatment type (Table 3).

### Immunologic status

All postoperative values (presented in percentage from baseline) for immunologic levels, as well as the mean values for POH12 and POH96 time intervals, are shown in Table 2.

As far as the immunologic status after colorectal surgery is concerned, serum IgG/IgA/IgM levels and circulating T/NK cells can be used as parameters for humoral immunity and cellular immunity, respectively.

### Humoral immunity status

**Figure 3:**
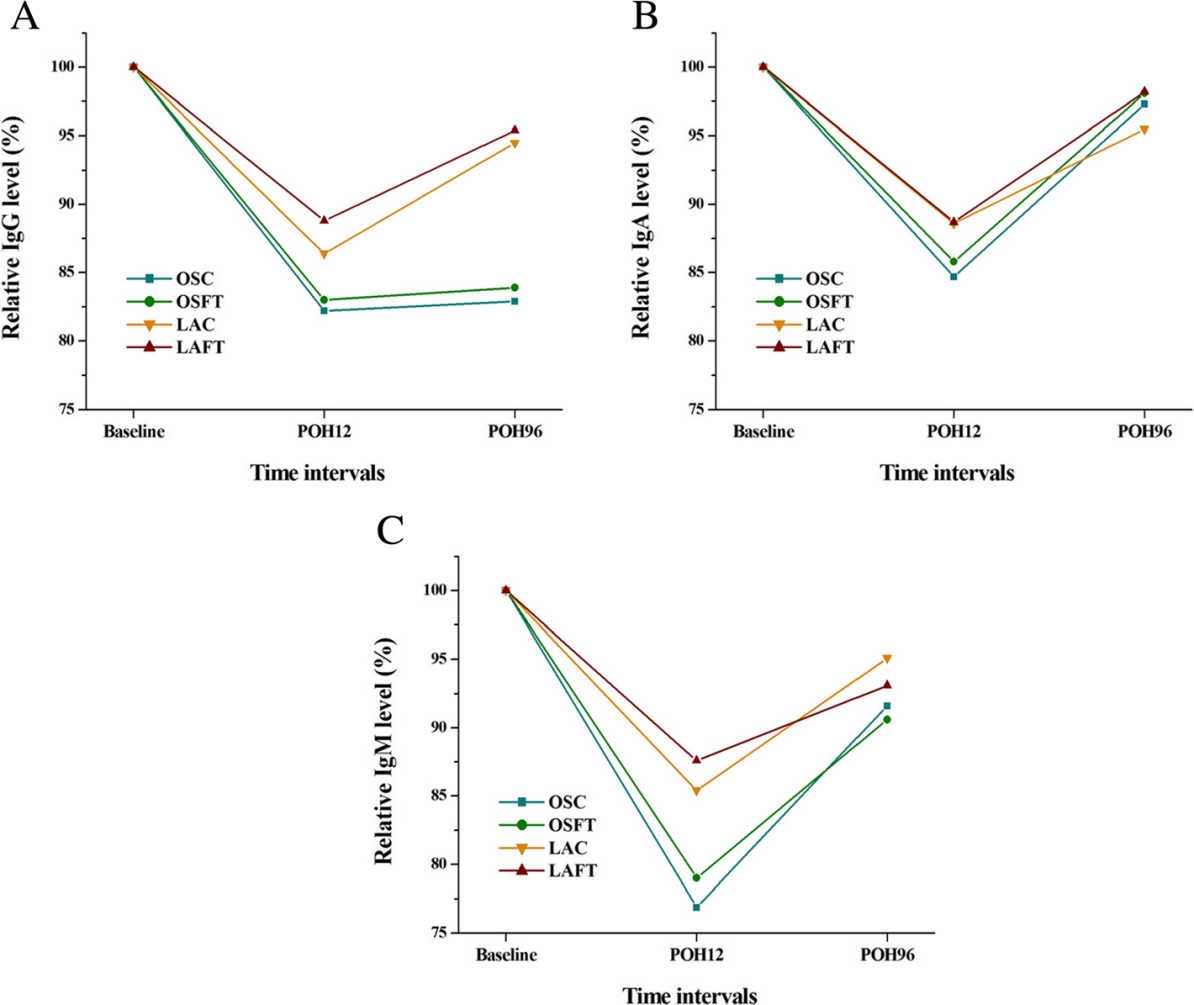
**Humoral immunity status of different time intervals in the four groups: IgG (A), IgA (B), IgM (C).**

Similar with albumin levels, postoperative IgG levels were also highest in the LAFT group and showed the similar rank with albumin levels in the four groups (Table 2; Figure 3A). Another surprising finding was that no matter the surgery type, little benefits of IgG level can be obtained from the fast-track care for the IgG levels were almost similar in same surgery-type groups with or without fast-track care. Repeated measures (two-way ANOVA) also indicated that the difference of IgG can be attributed to surgery type and not perioperative treatment (Table 3). No interaction was found between surgery type and perioperative treatment (Table 3).

IgA levels were highest in the LAFT group for both POH12 and POH96 time intervals, and IgM level was highest in the LAFT group only for POH12 intervals (Table 2; Figure 3B, C). But the following repeated measures (two-way ANOVA) revealed that no difference can be found between surgery type as well as perioperative treatment type (Table 3).

### Cellular immunity status

**Figure 4:**
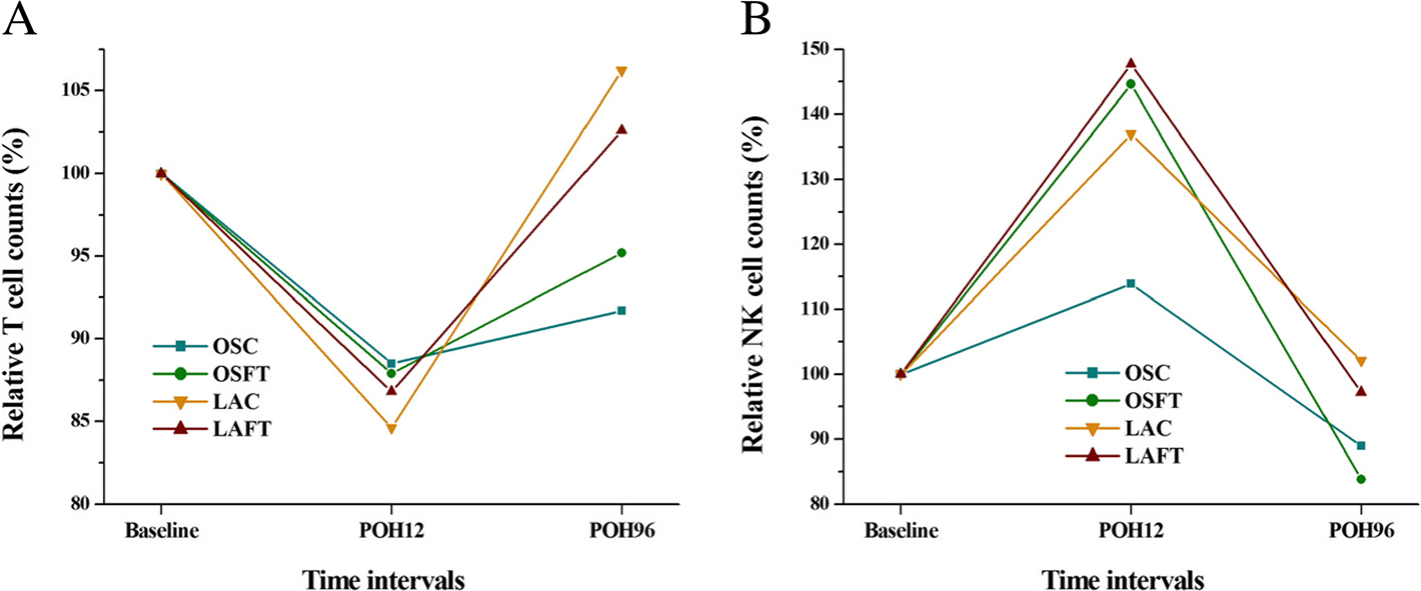
**T (A) and NK cell (B) counts of different time intervals in four groups.**

T and NK cell counts of POH 96 (Figure 4A, B) were higher in laparoscopy-included groups (LAC and LAFT) than that in open surgery-included groups (OSC and OSFT). But the following repeated measures (two-way ANOVA) revealed that no difference can be found between surgery type as well as perioperative treatment type (Table 3).

### Operative data and postoperative complications

The laparoscopic surgery-included groups (LAFT and LAC) exhibit significant differences of longer operation time, lower blood loss during operation, and shorter incision length when compared with open surgery-included groups (OSFT and OSC).

During 3-month follow-up, no differences in postoperative complication rates such as anastomotic leakage, ileus, and wound infection were observed between the groups (Table 1).

## Discussion

To the best of our knowledge, the exact role of laparoscopic surgery or fast-track care on both cellular or humoral immunity and postoperative nutrition still remains unclear to date. In clinical practice, fast-track surgery (FTS) adopts multiple perioperative treatments to attenuate the surgical stress response and thus accelerates postoperative recovery. The adoption of laparoscopic colorectal surgery came almost coupled with the introduction of FTS. Previous studies evaluating laparoscopic colorectal cancer surgery showed benefits of lower morbidity, reduced postoperative pain, and shortened hospitalization stay [16,17] without comprising long-term oncological outcomes as compared to open surgery [2,3].

So, fast-track perioperative care and mini-invasive surgery are both increasingly preferred in the treatment of colorectal cancer. Many clinical trials [2,3] and meta-analyses [4,18] had repeatedly shown the benefits of fast-track perioperative care or laparoscopic surgery for their short-time outcomes in reducing overall hospital stay with enhanced recovery of gastrointestinal and pulmonary function and less surgical stress. But most of the above trials only concerned on the clinical influences of the fast-track care and laparoscopic surgery, respectively; extremely few studies were designed to explore the biochemical impacts of the two counterparts on human immunologic and nutritional status. The aim of the present RCT was to evaluate prospectively the nutritional and immunologic status of the patients in four groups.

We are concerned for a long time about the combined treatment of laparoscopic colostomy and fast-track care [9,19]. On the 37th ESMO congress, we have reported the interim analysis of benefits from our FTMDT trial [20]; and here, we reported our updated and integrated data. We performed the present 2 × 2 randomized study of patients undergoing either open or laparoscopic surgery, combined with fast-track perioperative care or conventional treatment. Our results showed that combined laparoscopic surgery with fast-track care provided best postoperative recovery of nutritional and immunologic status as compared to each treatment modality alone. And the further two-way ANOVA statistic analysis helped us to distinguish the exact role and weight of the two counterparts: the earlier recovery of albumin; TRF and IgG levels can be attributed to surgery type and not perioperative treatment (*P* < 0.05).

Albumin, prealbumin (transthyretin), and transferrin are commonly called as “hepatic proteins” that are synthesized in the liver and to be used to evaluate nutritional status. Among the three proteins, Serum albumin has the longest half-life at 18 to 20 days and is most extensively used as a parameter of nutritional status and as an indicator of nutritional response to perioperative treatments [10–13].

What is most interesting in our present study was that, only in the laparoscopy-included groups (LAFT and LAC groups), the albumin level of POH96 was higher than that of POH12, indicating that laparoscopic surgery accelerated postoperative albumin level rising again and promoted the recovery of postoperative nutritional status. Further repeated measures (two-way ANOVA) indicated that the difference of albumin level can be attributed to surgery type and not perioperative treatment. That is, fast-track treatment retarded the drop of postoperative albumin level but could not accelerate the albumin level rising again. Of course, combined laparoscopic surgery with fast-track treatment provided best postoperative recovery of nutrition status. Several recent meta-analyses literatures have indicated that low serum albumin level was also an independent risk factor for the onset of postoperative complications and poorer surgical outcome [21,22]. So, the acceleration of postoperative albumin level recovery might be translated to the benefits of improved survival and better outcome.

Why another two hepatic proteins—prealbumin and transferrin—were not so outstanding and persistent with albumin in our present study? We assume two possible reasons for this question: the first is that serum transferrin level is determined not only by the nutritional status but also by the serum iron status, and it can be considered as a parameter of nutritional status only in the setting of normal iron level; secondly, prealbumin has the shortest half-life of 2–3 days. The synthesis of prealbumin can be easily influenced by the surgical stress, abnormity of liver function, etc. Therefore, it is less helpful of the three hepatic proteins for evaluating overall nutritional status. According to recent research by Fujii [23], serum albumin is also superior to prealbumin for predicting short-term recurrence in patients with operable colorectal cancer, which is an interesting theme needed to be investigated in our FTMDT trial after long-time follow-up.

As far as the immunologic status are concerned, circulating T and NK cells (representing cellular immunity) and serum IgG/IgA/IgM levels (representing humoral immunity) are usually used as the studying parameters. Representing approximately 75% of serum immunoglobulins in humans, IgG is the most abundant antibody isotype found in the circulation, playing a major role in the immune response to resist malignant cells or infectious pathogens [24]. T cells (usually express CD3) kill their target cells by recognizing MHC molecules on the target-cell membrane and produce a variety of cytokines required for the activation of other functional cells in the immune response system. Natural killer cell destroys abnormal target cells that lack cell-surface MHC class I molecule, such as infected and malignant cells [25]. Wichmann et al. [15] have reported fast-track care after colorectal surgery resulted in better-preserved cell-mediated immunity (T cells, T-helper cells, natural killer cells) when compared with conventional care. Yang [14] revealed fast-track care improved postoperative humoral immunity (globulin, immunoglobulin G, and complement 4) after open colorectal surgery. In this study, the 2 × 2 randomized LAFA trial indicated that the immune status are quite different in patients undergoing laparoscopy or open surgery with or without a fast-track perioperative care, and the immunologic level of HLA-DR expression on monocytes in patients undergoing laparoscopy with fast-track care remained highest [7]. To the best of our knowledge, the exact effects of laparoscopic colorectal surgery on both human cellular and humoral immunity in the context of FTS have not been studied to date.

Our present study found the beneficial effects of better-preserved and earlier recovery of humoral immunity status (serum IgG level) after colorectal surgery in LAFT group patients. Repeated measures (two-way ANOVA) also indicated that the difference of IgG could be attributed to surgery type and not perioperative treatment. As for the cellular immunity status, mean T cells and NK cells counts were higher in laparoscopy-included groups (LAC and LAFT) than that in open surgery-included groups (OSC and OSFT). But the following repeated measures revealed that no difference can be found between surgery type as well as perioperative treatment. Many studies have reported that the presence of persistent postoperative circulating tumor cells was strongly correlated with a poorer disease-free and overall survival in colon cancer patients [26,27], while the better-preserved immunity function may protect against potential tumor cell growth and seeding [28,29]. So, it is most important to investigate if the better-preserved immunity function may reduce the occurrence of clinical cancer metastasis or recurrence during the further follow-up study.

Possible explanation for the better-preserved and earlier recovery of nutritional and immunologic status in laparoscopy-included groups may be the corresponding beneficial effects of small incision, reduce of pain, as well as the early stage physical exercise resulting from the above two reasons. While the fast-track treatment seems worked mainly through reducing the stress response of internal environment and it plays a secondary role peripheral to laparoscopy as for earlier recovery of nutritional and immunologic status after surgery, our biochemical data suggested for the first time the different weights of the two counterparts (laparoscopy and fast-track care) in the clinical context: laparoscopic surgery may play a more important role than fast-track treatment in the earlier postoperative recovery of nutritional and immunologic status.

## Conclusions

So, we can draw our conclusions that: 1) Laparoscopic surgery accelerates postoperative nutrition and humoral immune levels rise again while fast-track treatment retards the drop of postoperative nutrition and immune levels; 2) Statistically, the beneficial effects that appeared in postoperative nutrition and immune status are due to laparoscopy in excess of fast-track treatment; 3) Combined laparoscopic surgery with fast-track treatment provides the best postoperative recovery of nutrition and immune status. So among the colorectal patients, the mode of laparoscopic resection with fast-track care is the most optimal treatment, especially in the patients with immune suppression or malnutrition. If only conventional care can be carried out, laparoscopic surgery should be recommended to accelerate postoperative recovery.
